# Outer Membrane Vesicles of *Porphyromonas gingivalis* Elicit a Mucosal Immune Response

**DOI:** 10.1371/journal.pone.0026163

**Published:** 2011-10-14

**Authors:** Ryoma Nakao, Hideki Hasegawa, Kuniyasu Ochiai, Shogo Takashiba, Akira Ainai, Makoto Ohnishi, Haruo Watanabe, Hidenobu Senpuku

**Affiliations:** 1 Department of Bacteriology I, National Institute of Infectious Diseases, Tokyo, Japan; 2 Department of Pathology, National Institute of Infectious Diseases, Tokyo, Japan; 3 Department of Microbiology, Nihon University, Tokyo, Japan; 4 Department of Pathophysiology-Periodontal Science, Okayama University Graduate School of Medicine, Okayama, Japan; University of California, Merced, United States of America

## Abstract

We previously reported that mutation of *galE* in *Porphyromonas gingivalis* has pleiotropic effects, including a truncated lipopolysaccharide (LPS) O-antigen and deglycosylation of the outer membrane protein OMP85 homolog. In the present study, further analysis of the *galE* mutant revealed that it produced little or no outer membrane vesicles (OMVs). Using three mouse antisera raised against whole cells of the *P. gingivalis* wild type strain, we performed ELISAs to examine the reactivity of these antisera with whole cells of the wild type or the *galE* mutant. All three antisera had significantly lower reactivity against the *galE* mutant compared to wild type. OMVs, but not LPS, retained the immunodominant determinant of *P. gingivalis*, as determined by ELISAs (with wild type LPS or OMVs as antigen) and absorption assays. In addition, we assessed the capacity of OMVs as a vaccine antigen by intranasal immunization to BALB/c mice. Synthetic double-stranded RNA polyriboinosinic polyribocytidylic acid [Poly (I∶C)], an agonist of Toll-like receptor 3 (TLR3), was used as the mucosal adjuvant. Vaccination with OMV elicited dramatically high levels of *P. gingivalis*-specific IgA in nasal washes and saliva, as well as serum IgG and IgA. In conclusion, the OMVs of *P. gingivalis* have an important role in mucosal immunogenicity as well as in antigenicity. We propose that *P. gingivalis* OMV is an intriguing immunogen for development of a periodontal disease vaccine.

## Introduction

Periodontitis is an oral disease characterized by destruction of periodontal tissues and ultimately exfoliation of the teeth in humans [1]. In addition, recent reports from epidemiological studies [2], [3], [4] as well as *in vitro* and animal model experiments [5], [6], [7] have shown an association between periodontitis and systemic diseases such as diabetes mellitus, cardiovascular disease, and atherosclerosis. Therefore, development of a safe vaccine for periodontal diseases would be a great benefit to improving public health. Among the various Gram-negative anaerobes that reside within the subgingival pockets, *Porphyromonas gingivalis* is a major causative agent in the initiation and progression of severe forms of periodontal disease [1]. Several virulence factors of *P. gingivalis* are known, including fimbriae, gingipains, hemagglutinins, lipopolysaccharide (LPS), and outer membrane vesicles (OMVs) [1]. However, it is not known that *P. gingivalis* components are targeted by the protective humoral immune system during disease onset and progression.

Gram-negative bacteria are distinguished from other prokaryotes by an outer membrane that surrounds their peptidoglycan layer. The outer membrane contains essential molecules, such as LPS and outer membrane proteins [8]. Of particular medical interest are the miscellaneous surface-exposed molecules on intact bacteria that are recognized by the immune system and possess a powerful potential to activate the host immune system. Gram-negative bacteria release OMVs from the cell surface during bacterial growth [9]. OMVs range in size from 20 to 250 nm in diameter and contain not only components of the outer membrane, such as LPS, outer membrane proteins, and phospholipids, but also periplasmic proteins and cell wall components, i.e., peptidoglycan, given that OMVs entrap some of the underlying periplasmic proteins and small particles of the cell wall when the blebs are extruded from the cell surface. OMVs play a role in such pathogenic processes as toxin export [10], [11], [12] and adherence to eukaryotic cells [13]. As with OMVs of other bacteria, *P. gingivalis* OMVs contain several virulence factors, such as LPS, fimbriae, and gingipains [14], [15], [16], [17]. Recently, *P. gingivalis* has been shown to possess a system that selectively sorts virulence factors into OMVs [18]. OMVs of *P. gingivalis* also can be internalized into host cells via a lipid-raft-dependent endocytic pathway and are subsequently routed to the early endosome followed by sorting into lysosomal compartments [19]. After lysis of the OMV, various antigens may be recognized and processed by antigen-presenting cells such as dendritic cells and macrophages, leading to induction of adaptive immunity including pathogen-specific antibody production.

OMVs have also been recognized as a vaccine candidate for infectious diseases. In several countries, wild type OMVs of *Neisseria meningitidis* serogroup B were approved as vaccines for parenteral use with reported efficacy rates of 70% to 83% in adults and children [20]. Currently, OMV vaccines are the only formulation that have shown efficacy against serogroup B meningococcal diseases. Intranasal administration of OMVs derived from *Vibrio cholerae* has also induced protective immunity against this gastrointestinal pathogen in mice [21]. In the case of *P. gingivalis*, although parenteral administration of OMVs in mice is protective against challenge infection [22], the efficacy of OMVs as an intranasal vaccine has not yet been determined.

In the present study, we identified an OMV-negative mutant of *P. gingivalis* and investigated its antigenicity by comparative analysis with the wild type strain. Our results indicated that OMVs play a significant role in the antigenicity of *P. gingivalis*. In addition, we demonstrated that intranasal administration of *P. gingivalis* OMVs effectively elicited not only serum IgG and IgA, but also secretory IgA (s-IgA) in nasal washes and saliva that recognize *P. gingivalis*.

## Materials and Methods

### Bacterial strains and culture conditions

*P. gingivalis* ATCC 33277 and the *galE* mutant [23] were maintained in brain heart infusion (BHI) broth supplemented with hemin and menadione (HM) or on BHI-HM blood agar plates in an anaerobic chamber (miniMACS anaerobic workstation, Don Whitley Scientific Ltd., Shipley, UK) using 80% N_2_, 10% H_2_, and 10% CO_2_.

### Preparation of OMVs and LPS from *P. gingivalis*

Preparation of OMV was performed as described previously with some modifications [11]. In brief, the supernatant of a two-day culture of strain 33277 was collected by centrifugation at 3,410× *g* for 15 min at 4°C, then filtered through a 0.22-µm PVDF filter and ultra-centrifuged at 100,000× *g* for three hours at 4°C in a 41 Ti rotor (Beckman Instruments, Inc., USA). The resulting OMV pellet was resuspended in 20 mM Tris-Cl (pH 8.0) and the protein concentration was measured by Bradford assay [24] using bovine serum albumin as a standard. Preparation of LPS from *P. gingivalis* was performed using the hot phenol water extraction method with some modifications [23]. Finally, LPS was lyophilized, weighed and used for mitogenic assays or ELISAs.

### Mitogenic assays

The mitogenic activity of LPS prepared from the wild type and the *galE* mutant was assessed by adding various concentrations of LPS (0.1–100 µg/ml) to primary splenocyyte cultures (5×10^5^ cells/ml) prepared from BALB/c mice. A proliferation assay using WST-1 (Dojindo Laboratories, Kumamoto, Japan) was then performed.

### Scanning electron microscopy (SEM)

To analyze cell morphology, *P. gingivalis* wild type or the *galE* mutant strain were grown on non-treated plastic sheets (Wako Chemical Ltd., Osaka, Japan) placed in 6-well polystyrene cell culture plates. Four ×10^7^ CFU of *P. gingivalis* were grown in two ml of BHI-HM broth per well at 37°C for 12 hours under anaerobic conditions. For analysis of OMVs derived from *P. gingivalis*, the bacterial supernatant was collected at different time points, then incubated on poly L-lysine-coated cover slips for 30 min at room temperature to attach the OMVs to the glass. The attached bacteria and OMVs were fixed with 2.5% glutaraldehyde and 2% paraformaldehyde in PBS for 30 min at room temperature, followed by three washes in PBS. The samples were washed in PBS; dehydrated in 50% ethanol to absolute ethanol; immersed in isoamyl acetate; dried by critical point drying; coated with osmium vapor using an osmium plasma coater; and visualized by SEM (S-5200, HITACHI, Hitachi, Japan).

### Time-course analysis of bacterial growth and LPS in the supernatant

Growth assays were performed by measuring OD_600_. The bacterial supernatant was collected by centrifugation at 17,400× *g* for two minutes at different time points. The amount of LPS in the supernatant was measured by Limulus amoebocyte lysate (LAL) reagent according to the manufacturer's instructions (Seikagaku Corporation, Tokyo, Japan).

### Immunization

All animal experiments were performed in accordance with our institutional guidelines. Female BALB/c mice (Japan SLC, Inc., Hamamatsu, Japan), aged 6 to 8 weeks at the time of immunization, were used in all experiments. For the first half of the experimental plan, the mice were immunized by the conventional method to obtain *P. gingivalis*-specific antisera as follows: 1×10^8^ CFU of heat-killed bacteria emulsified in TiterMax Gold (TiterMax USA; Norcross, GA) was injected into mice intraperitoneally followed by two booster injections on days 8 and 15 with 5×10^7^ CFU of heat-killed bacteria in incomplete Freund's adjuvant. For the second half of the experimental plan, the mice were immunized intranasally. In brief, mice were immunized by dropping 5 µl of PBS containing 5 µg of polyriboinosinic polyribocytidylic acid [Poly (I∶C)] (Sigma, St. Louis, MO) with or without 0.5 µg of immunogen (OMVs or heat-killed whole cells of *P. gingivalis* ATCC 33277) into each nostril on day 0 and week 3 under anesthesia. The protein concentration of these immunogens was determined by Bradford assay [24]. At two weeks after the second immunization, sera, nasal washes, and saliva specimens were collected and used for ELISA. Saliva samples were obtained as described previously [25].

### ELISA

Reactivities of mouse sera and nasal washes with whole cells of the bacterial strains were tested using an ELISA described previously with some modifications [26]. Unwashed whole cells of *P. gingivalis* were prepared from fresh bacterial culture by centrifuging only once at 3,410× *g* for 15 minutes at 4°C without washing. For washed whole cells, bacteria were collected by centrifugation and resuspended in PBS by pipetting. The centrifugation and washing steps were repeated. Both the unwashed and washed bacteria were collected and freeze-dried. The dried bacteria were weighed and used as antigen for whole-cell ELISA. ELISA plates were coated with 10 µg of the freeze-dried bacteria resuspended in 100 µl of ELISA coating buffer per well. After overnight blocking at 4°C with 1% skim milk in PBS with 0.5% Tween 20 (PBS-T), each serum or nasal wash sample was serially diluted with 1% skim milk in PBS-T, added to the wells and incubated for 1 hour at 37°C. The wells were then incubated for 1 hour at 37°C with alkaline phosphatase-conjugated goat anti-mouse IgG, IgA, and IgM (Invitrogen, Carlsbad, CA) at a dilution of 1∶1000. Subsequently, *P. gingivalis*-specific antibody was detected by chromogenic development using para-nitrophenyl phosphate as the alkaline phosphatase substrate. Absorbance at OD_405_ was measured at different time points; 15 minutes, 30 minutes, 1 hour, and 2 hours. The titer of each antiserum recognizing LPS and OMV from *P. gingivalis* 33277 was determined using the same ELISA protocol, except the microtiter wells were initially coated with antigen at 100 ng/well. In the absorption assay, each serum sample was diluted at 1∶1000 in PBS and pre-incubated with 10 ng/ml of LPS or OMV purified from strain 33277 for 1 hour at 37°C. Then, the absorbed serum samples were passed through a Detoxigel™ (Thermo Fisher Scientific, Waltham, MA) column twice to remove possible contaminant LPS and LPS-associated molecules. An LAL assay was performed to confirm that the serum did not contain any detectable LPS. The purified sera were used for ELISA.

### Statistical analysis

Statistical analysis was performed using the Mann-Whitney's U-test. P-values of 0.05 or less were considered to be statistically significant.

## Results

### *P .gingivalis galE* mutant essentially lacks OMVs as well as LPS in the supernatant

We previously demonstrated that mutation of the *galE* gene in *P. gingivalis* caused pleiotropic effects associated with the metabolic pathway of galactose, resulting in autoaggregation/biofilm formation as well as deglycosylation of LPS and the outer membrane protein Omp85 homolog [23], [27]. Notably, *galE* mutation also affects components of the outer membrane, therefore we hypothesized that morphological changes might occur at the bacterial surface of the *galE* mutant.

**Figure 1:**
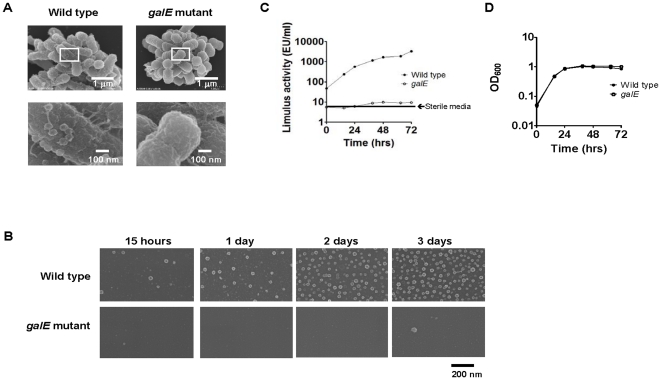
SEM images of *P. gingivalis* wild type and the *galE* mutant. (A) *P. gingivalis* wild type and *galE* mutant were grown on plastic sheets for 12 hours. Upper panels show the morphology of the attached cells. The lower panels, enlargements of the squares in the upper panels, show the presence and absence of OMVs on wild type and *galE* mutant cells, respectively. The scale is shown at the lower right of each electron micrograph. (B, C, and D) OMVs of wild type and the *galE* mutant were harvested at different time points during culture. (B) The OMVs were applied to plastic sheets, and visualized by SEM. The scale is shown at the lower right. The limulus activity (C) and growth (D) of each sample were recorded.

Firstly, we examined the morphology of wild type and the *galE* mutant using scanning electron microscopy (SEM). There was no difference in the size or shape of mutant cells compared to wild type (Fig. 1A). On the surface of the wild type cells, many OMVs were clearly visible as spherical structures approximately 50–70 nm in diameter (Fig. 1A). However, we could not find any OMVs on the surface of the *galE* mutant (Fig. 1A). We also analyzed by SEM the OMVs discharged into the bacterial supernatant at different time points during three days of culture (Fig. 1B). The numbers of OMVs in the supernatant of the wild type strain increased until day 3 (Fig. 1B). In contrast, we could not find any OMVs in the supernatant of the *galE* mutant throughout the three days (Fig. 1B). To test whether the *galE* mutant also releases less LPS during culture, we examined the kinetics of the LPS activity in the supernatants of the wild type and *galE* mutant by LAL assay (Fig. 1C) with their growth curves (Fig. 1D). The Limulus activity in the supernatant of the wild type increased through day 3. In contrast, the Limulus activity in the *galE* mutant supernatant increased minimally during the same time period, remaining similar to that of sterile BHI-HM liquid media (7.54 EU/ml) shown by an arrow in Fig. 1C. To evaluate LPS quality, we compared LPS prepared from the wild type and the *galE* mutant in a mitogenic assay. The mitogenic assay showed that LPS from both strains had similar activities (data not shown), suggesting that the lower Limulus activity of *galE* mutant supernatant compared to wild type (Fig. 1C) is due to less total LPS, not lower LPS activity.

### OMVs of *P. gingivalis* retain the immunodominant determinants

**Figure 2:**
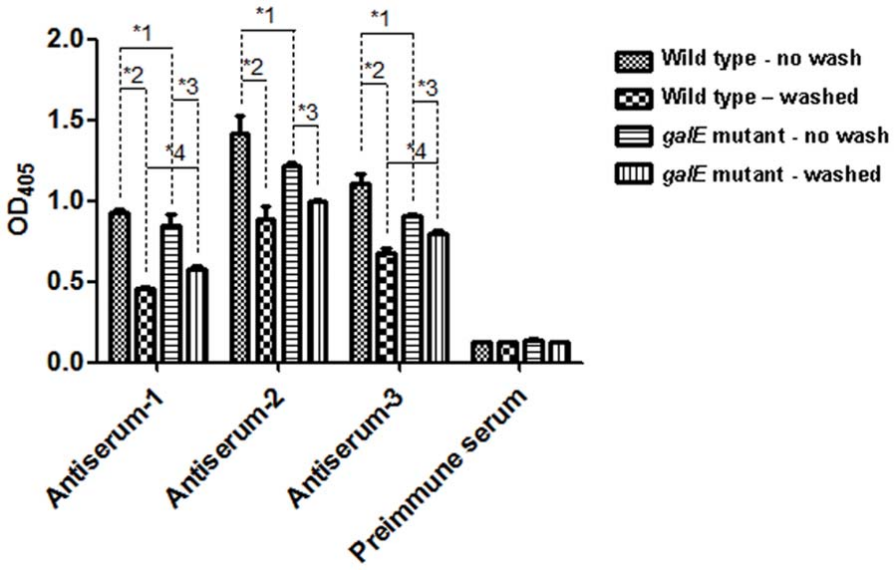
Analysis of antigenicity of whole cells from wild type and the *galE* mutant using *P. gingivalis* antisera. ELISA plates were coated with freeze-fried *P. gingivalis* wild type or the *galE* mutant. Bacteria were either washed twice with PBS or left unwashed before coating. *P. gingivalis* antisera from three different mice and a pre-immune serum were used at dilutions of 1∶1,000. Sera reactivity was determined as the absorbance at 405 nm (mean ± SD) for triplicate assays after a 30-min incubation with alkaline phosphatase substrate. Asterisks-1, -2, -3, and -4 denote statistically significant differences (*p*<0.05).

We next examined whether OMVs function as a reservoir of immunoreactive antigens. We performed whole-cell ELISA to test the reactivity of preimmune serum and three mouse antisera (raised against whole *P. gingivalis* wild type by conventional immunization) to the wild type and *galE* mutant. All three antisera showed a significantly higher reaction to unwashed wild type than to unwashed *galE* mutant (asterisk-1 in Fig. 2), while background reactivity of preimmune serum to the wild type and *galE* mutant were comparable, irrespective of washing. All antisera showed significantly lower reactivities to both washed wild type and the washed *galE* mutant, compared to when the corresponding bacteria without washing served as ELISA antigen (asterisk-2 and -3 in Fig. 2). Washing resulted in a larger decrease in antisera reactivity for the wild type (asterisk-2 in Fig. 2) than for the *galE* mutant (asterisk-3 in Fig. 2), indicating that cell washing drastically reduced surface antigenicity due to loss of OMVs and probably other bacterial appendages as well. When washed cells were used as antigen, two of the three antisera, antiserum-1 and -3, had significantly stronger reactivity to the *galE* mutant than to the wild type (asterisk-4 in Fig. 2). It is possible that the *galE* mutant is more antigenic than the wild type, because antigenic determinants on the outer membrane of the *galE* mutant may be more readily exposed to the environment by deglycosylation of LPS [23] and/or outer membrane glycoproteins [27].

**Figure 3:**
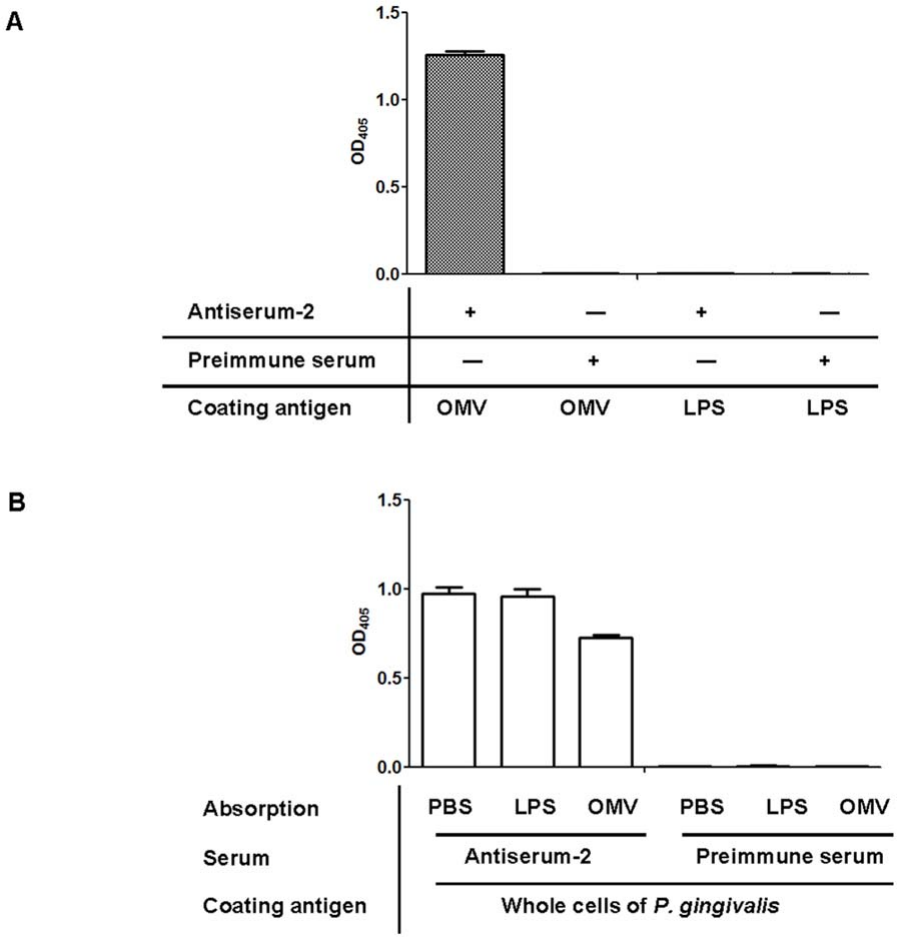
*P. gingivalis* antiserum cross-reacts strongly with OMVs, but not with LPS. (A) ELISA plates were coated with LPS and OMVs of *P. gingivalis*. *P. gingivalis*, antiserum-2 and pre-immune serum were used at dilutions of 1∶1,000. Sera reactivity was determined as the absorbance at 405 nm (mean ± SD) for triplicate assays after a 30-min incubation with alkaline phosphatase substrate. (B) To test whether antiserum-2 cross-reacts with OMVs or LPS, we absorbed the serum against LPS and OMVs and removed bound antibodies. ELISA results are expressed as absorbance at 405 nm (mean ± SD) after a 30-min incubation with alkaline phosphatase substrate.

In Figure 2, we showed that OMVs associated with bacteria enhanced antigenicity. However, LPS may also play a key role in eliciting antibody production and therefore may affect the antigenicity of Gram-negative bacteria. To determine whether LPS and/or OMVs are involved in antigenicity, we examined the reactivity of mouse serum IgG to LPS and OMVs using preimmune serum and one of three antisera against *P. gingivalis* that showed reactivity to the whole cells, antiserum-2 (Fig. 2). The reactivity of preimmune serum against OMV or LPS was low (Fig. 3A). Antiserum-2 reacted strongly to OMV, but not to LPS (Fig. 3A).

To provide further confirmation, we performed an absorption assay where specific antibodies were absorbed from the sera before ELISA. To remove LPS- or OMV- specific antibodies, the sera were absorbed by pre-incubation with LPS or OMV. The reactivity of antiserum-2 to whole *P. gingivalis* cells decreased significantly after pre-incubation with OMV in comparison to pre-incubation with PBS (Fig. 3B). However, pre-incubation with LPS did not influence the reactivity of the antiserum-2 against *P. gingivalis* (Fig. 3B). We also obtained similar results obtained by absorption assay using the other antisera (antiserum-1 and -3).

### OMVs of *P. gingivalis* elicit *P. gingvalis*-specific humoral immune responses

**Figure 4:**
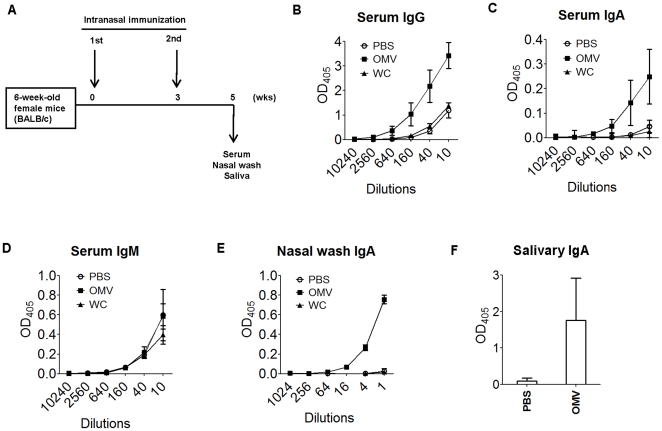
Immunogenicity of OMVs and whole cells of *P. gingivalis* after intranasal immunization. (A) The timeline of immunization is shown. (B–E) ELISA plates were coated with *P. gingivalis* whole cells. Samples of serum, nasal washes and saliva taken from mice immunized with *P. gingivalis* OMV, *P. gingivalis* whole cells (WC), and sham-immunized (PBS) mice. *P. gingivalis*-specific serum IgG (B), serum IgA (C), serum IgM (D), and nasal wash IgA (E) were examined by ELISA. For the salivary IgA (F), un-diluted saliva samples from OMV-immunized mice were compared with those from sham-immunized (PBS) mice. The results of triplicate assays are expressed as absorbance (mean ± SD) at 405 nm after a 30-min incubation with alkaline phosphatase substrate. In (A) to (E), the serum and nasal wash samples were from four mice per OMV-immunized group, four mice per *P. gingivalis* whole cells-immunized group (WC) and two mice per PBS control group. In (F), the saliva samples were from two PBS-immunized mice and three OMV-immunized mice.

To investigate whether OMVs have the potential to induce not only antibodies in blood, but also mucosal antibodies that recognize *P. gingivalis* in mice, we designed an intranasal immunization protocol using OMVs and a mucosal adjuvant (Fig. 4A). Double-stranded RNA has been shown to be an effective adjuvant for mucosal vaccination against influenza virus [28], [29]. Therefore, we chose Poly (I∶C), a double-stranded RNA adjuvant, as our vaccine adjuvant. Briefly, after intranasal immunization of OMVs or whole-cell *P. gingivalis* twice on day 0 and day 21, mice were sacrificed at week five, and Ig titers were determined by whole cell ELISA. Intranasal immunization with *P. gingivalis* whole cells did not effectively induce *P. gingivalis*-specific antibodies (Fig. 4B–E). In contrast, immunization with OMVs strongly induced *P. gingivalis*-antibodies in mice (Fig. 4B, C, and E). Notably, OMVs also strongly induced nasal wash IgA, as well as serum IgG and IgA. As with the nasal wash, we also observed strong induction of saliva IgA in mice immunized with OMVs (Fig. 4F), but not in either sham-immunized (PBS) (Fig. 4F) or pre-immune mice (data not shown). *P. gingivalis*-specific serum IgM was not found due to similar reactivity among all mouse groups (Fig. 4D).

## Discussion

In Fig. 1A and B, we demonstrated that OMVs were not detectable in the *galE* mutant. Growth of the *galE* mutant was similar to that of the wild type (Fig. 1D), however, the limulus activity of the respective supernatants was quite different. The limulus activity of the wild type strain supernatant increased steadily through culture day 3, while that of the *galE* mutant remained similar to baseline (Fig. 1C). These data suggest that OMV probably plays an important role in dissemination of LPS to the external environment during growth. However, since *galE* mutation causes pleiotropic effects [23], [27], it is also possible that changes in OMV formation and LPS release are two unrelated events in this mutant.

When washed bacterial cells were used as antigen for ELISA, all *P. gingivalis* antisera exhibited drastically decreased reactivity to the wild type (asterisk-2 in Fig. 2), but only mildly decreased reactivity to the *galE* mutant (asterisk-3 in Fig. 2). *P. gingivalis* antiserum recognized OMV, but not LPS (Fig. 3A). Absorption assays revealed that *P. gingivalis* antiserum reactivity to whole bacteria decreased after pre-incubation with OMVs (Fig. 3B). Our data suggest that OMVs play a pivotal role in the antigenicity of *P. gingivalis* and other appendages loosely tethered to the outer membrane, such as fimbriae may also affect the antigenicity of *P. gingivalis*.

The presence of OMVs on *P. gingivalis* (Fig. 1A) may confer increased antigenicity simply because the vesicles effectively expand the bacterial surface area. On the other hand, surprisingly, *P. gingivalis*-specific antibody was not detectable when mice were immunized with whole *P. gingivalis* cells, while OMV immunization strongly elicited specific antibodies (Fig. 4). Therefore, an alternative reason that both antigenicity and immunogenicity were enhanced by the presence of OMVs might be that immunodominant determinants are more concentrated on OMVs than on the bacterial surface itself. Many reports have shown that virulence factors are associated with OMVs in Gram-negative bacteria (reviewed by [30]), including *P. gingivalis* [14], [15], [16], [17], [18]. Thus, we suggest that our strategy of OMV vaccination via the nasal cavity might be applicable to *P. gingivalis* bacterial infections whose virulence factors are enriched in the OMV.

Double-stranded RNA, such as the Poly (I∶C) and Ampligen®, is a Toll-like receptor 3 (TLR3) agonist. Promising results have been obtained using Poly(I∶C) or Ampligen® as an adjuvant in flu vaccine delivered intranasally to mice [28], [29]. The safety of Ampligen® also has been established in clinical trials for patients with chronic fatigue syndrome in the U.S. [31]. On the other hand, in many animal studies cholera toxin (CT) B subunit or the mutant CTB [32] has been used as a strong adjuvant to induce protective immunity. However, use of heat-labile enterotoxin (LT), which is structurally and functionally similar to CT, has been linked to severe complications, such as several cases of Bell's palsy (facial paralysis) [33]. Therefore, at present, an adjuvant derived from a toxin is impractical for use in a human vaccine, especially for periodontal disease vaccine, because the benefit of the vaccine must far outweigh the risk of serious side effects.

In this study, we applied Poly (I∶C) as an adjuvant for OMV intranasal immunization of mice. Without using a toxin-derived adjuvant, we successfully elicited an s-IgA response in saliva as well as a serum IgG response. In periodontal pockets, periodontopathic bacteria float as planktonic cells or form biofilms in the fluid composed of gingival cervicular fluid (GCF) and saliva. While the GCF contains abundant immunoglobulins (mostly IgG) exuding from blood vessels, the saliva contains abundant s-IgA. Therefore, both the systemic and mucosal immune responses contribute to humoral immunity in the oral cavity and are important in the context of a vaccine strategy against periodontal diseases. In particular, s-IgA is regarded as a main player in immunological defense at the mucosal surface because pathogen-specific s-IgA can inactivate the pathogen before it invades the host. In addition, s-IgA is generally more cross-reactive against pathogen variants than IgG and other classes of immunoglobulins.

It has been reported that intraperitoneal administration of OMVs derived from *Salmonella typhimurium* activated *Salmonella*-specific T and B cell responses and elicits protective immunity against challenge with live bacteria in mice [34]. A recent report showed that intranasal administration of OMVs derived from *V. cholerae* successfully induced protective immunity in mice [21], although it remains unknown whether undesirable molecules such as CT are present as containants in the OMV preparation and whether clinical use is safe. As OMV is a cell-free antigen, its use as a vaccine is safer than the conventional live-attenuated vaccine. In addition, an OMV vaccine is superior to other formulations, such as a purified protein vaccine, for economical reasons and in terms of its stability at ambient temperature. In the present study, we characterized the immunological properties of *P. gingivalis* OMV. In conclusion, we suggest that *P. gingivalis* OMV might have application as a periodontal disease vaccine. To our knowledge, this is the first study using a combination of bacterial OMV and Poly (I∶C) for strong induction of bacterial-specific s-IgA in saliva and nasal washes as well as IgG and IgA in serum. Further studies will be required to examine whether this strategy can protect against bacterial challenge and to elucidate the mechanism of humoral immune responses to intranasal administration of OMV.
